# Treatment prescribing patterns in patients with juvenile idiopathic arthritis (JIA): Analysis from the UK Childhood Arthritis Prospective Study (CAPS)^☆☆^^★^

**DOI:** 10.1016/j.semarthrit.2016.06.001

**Published:** 2016-10

**Authors:** Rebecca Davies, Roberto Carrasco, Helen E. Foster, Eileen M. Baildam, S.E. Alice Chieng, Joyce E. Davidson, Yiannis Ioannou, Lucy R. Wedderburn, Wendy Thomson, Kimme L. Hyrich

**Affiliations:** a Arthritis Research UK Centre for Epidemiology, Centre for Musculoskeletal Research, Manchester Academic Health Science Centre, The University of Manchester, Manchester, UK; b Musculoskeletal Research Group, Institute Cellular Medicine, Newcastle University and Paediatric Rheumatology, Great North Children׳s Hospital, Newcastle upon Tyne, UK; c Department of Paediatric Rheumatology, Alder Hey Children׳s Hospital NHS Foundation Trust, Liverpool, UK; d Department of Rheumatology, Royal Manchester Children׳s Hospital, Manchester, UK; e Department of Paediatric Rheumatology, Royal Hospital for Children, Glasgow, UK; f Arthritis Research UK Centre for Adolescent Rheumatology, University College London, London, UK; g Infection, Inflammation, and Rheumatology Section, Institute of Child Health, UCL and Great Ormond Street Hospital NHS Trust, UCL Institute of Child Health, London, UK; h Arthritis Research UK Centre for Genetics and Genomics, Centre for Musculoskeletal Research, Manchester Academic Health Science Centre, The University of Manchester, Manchester, UK; i NIHR Manchester Musculoskeletal Biomedical Research Unit, Central Manchester University, Hospitals NHS Foundation Trust and University of Manchester Partnership, Manchester, UK

**Keywords:** Juvenile idiopathic arthritis, Biologic therapy, DMARD therapy, Treatment

## Abstract

**Objective:**

Initial treatment of juvenile idiopathic arthritis (JIA) is largely based on the extent of joint involvement, disease severity and ILAR category. The licensing of biologic therapies for JIA has expanded treatment options.

The aims of the study are (1) to describe treatment prescribing patterns in JIA over the first 3 years following first presentation to paediatric rheumatology and (2) to determine whether patterns of treatment have changed as biologics have become more widely available.

**Methods:**

Children with at least 3 years of follow-up within the Childhood Arthritis Prospective Study (CAPS) were included.

For analysis, children were placed into one of five groups according to their initial presentation to paediatric rheumatology: oligoarthritis (oJIA), polyarthritis (pJIA), systemic (sJIA), enthesitis-related arthritis (ERA) and psoriatic arthritis (PsA). Treatment patterns over 3 years were described.

**Results:**

Of 1051 children, 58% received synthetic disease-modifying anti-rheumatic drugs (sDMARD) and 20% received biologics over the 3 years. Use of sDMARDs and biologics was higher in more severe disease presentations (sJIA and pJIA); however, 35% and 10% who presented with oJIA were also treated with sDMARDs and biologics, respectively. The number of children receiving sDMARD after 2006 was higher (*p* = 0.02); however, there was no difference in biologic prescribing before and after 2006 (*p* = 0.4).

**Conclusions:**

A high proportion of children presenting with JIA received sDMARDs plus/minus biologics during 3 years of follow-up. This was most common for patients with severe JIA but was also prescribed for patients with oligoarticular disease, despite the lack of evidence for effectiveness in this category.

## Introduction

Juvenile idiopathic arthritis (JIA) is the most common chronic, inflammatory rheumatic disease among children with a UK prevalence rate of approximately 1 in 1000 [1]. JIA is an umbrella term for a heterogeneous group of conditions, classified according to the International League of Associations for Rheumatology (ILAR), each with its own distinctive characteristics [2]. Initial treatment is largely based on the extent of joint involvement, disease severity and category [3]. Typically intra-articular steroid injections are given as first-line treatment to patients presenting with oligoarthritis (defined as four or less joints involved), while synthetic disease-modifying anti-rheumatic drugs (sDMARDs) such as methotrexate are indicated as first-line treatment for systemic and polyarticular presentations [3].

There remain a proportion of children who will not respond to these initial therapies and further interventions will be required [4]. For others, their category may evolve, such as an initial oligoarticular presentation developing into a polyarticular course, resulting in a stepup in therapy. The introduction of biological agents in the last 15 years has offered further treatment options to those with severe polyarticular or systemic disease who do not respond adequately to initial therapy. However, the data regarding the proportion of children in routine care who eventually require biologics are generally lacking. Etanercept is the most commonly prescribed biologic in JIA and has been shown to be efficacious and safe in a number of studies [5], [6], [7], [8]. More recently, the evidenced based choice of biologics for JIA has expanded to include other anti-TNF therapies [e.g., adalimumab and infliximab (off-label use in Europe)] as well as alternative cytokine blockade for both systemic and polyarticular JIA (e.g., the IL-6 inhibitor tocilizumab and IL-1 blockade with anakinra) [9].

Treatment options for children with persistent oligoarthritis, which does not respond to intra-articular steroid injections are less clearly defined as these children are not often included in clinical trials, although many will be prescribed systemic therapies. The extent to which this occurs is not well described, although a recent Canadian study reported that patients presenting with oligoarthritis have a 40% chance of receiving sDMARDs and a 6.6% chance of receiving a biologic over 5 years [10].

The primary aim of this analysis is to describe the patterns of arthritis treatment prescribed among an inception cohort of children over the first 3 years following first presentation to paediatric rheumatology with JIA. The second aim is to look at whether the patterns of treatment have changed in more recent years as biologic therapies have become more widely available.

## Patients and methods

### Patients

Children included in this study are participants in the Childhood Arthritis Prospective Study (CAPS), a prospective inception cohort study of patients with JIA established in 2001 [11]. Children <16 years with new onset arthritis in one or more joints lasting at least 2 weeks are recruited from one of 7 UK rheumatology centres within 6 months of first presentation to paediatric rheumatology.

The study was approved by the UK Northwest Multicentre Research Ethics Committee. Written informed consent is obtained from the parent(s)/guardian for all participant children and children, if considered able, provide assent.

### Data collection and follow-up

Following consent, data are captured from baseline (first presentation to paediatric rheumatology) by a clinical research nurse using a standardised questionnaire, which captures information including demographics and disease duration. Information from clinical notes is also obtained including a physician-assigned ILAR (where applicable), clinical markers of disease and a list of current medication. JIA core outcome variables [12] are also collected including active and limited joint counts, 10 cm visual analogue scale (VAS) physician global assessment, 10 cm VAS parent assessment of wellbeing, erythrocyte sedimentation rate (ESR), the Childhood Health Assessment Questionnaire (CHAQ) and a 10 cm visual analogue scale (VAS) pain score.

Patients are followed annually for 5 years, then at 7 and 10 years. At each follow-up visit, further data on current disease activity, ILAR classification, and changes to medications are captured.

### Statistical analysis

This analysis included all children with a physician diagnosis of JIA recruited up to January 1, 2011 to allow all children the opportunity to contribute at least 3 years of follow-up data by January 1, 2014, the data cut-point.

Children were categorised into one of five disease presentations according to the number of active joints at first presentation to paediatric rheumatology (study baseline) and their initial physician-assigned ILAR category (within the first 6 months following presentation): oligoarthritis (oJIA), polyarthritis (pJIA), systemic (sJIA), enthesitis-related arthritis (ERA) and psoriatic arthritis (PsA). Patients were classified into each category as follows—oJIA: <5 active joints at first presentation and an initial ILAR category of persistent oligoarthritis or undifferentiated JIA (uJIA) with <5 active joints, pJIA: ≥5 active joints at first presentation and an initial ILAR category of rheumatoid factor (RF) positive or negative polyarthritis, uJIA with ≥5 active joints, or extended oligoarthritis, SJIA: initial ILAR category of systemic JIA, ERA: initial ILAR category of ERA and PsA: initial ILAR category of PsA.

Children were excluded if they withdrew consent from the study, had a missing ILAR category or joint count at baseline and 6 months, or were reclassified as not having JIA.

Treatment exposures over the first 3 years following presentation to paediatric rheumatology were determined and categorised into steroids [intra-articular, oral or intravenous (no intramuscular steroids were recorded in this group)], sDMARDs and biologics. The use of non-steroidal anti-inflammatory drugs (NSAIDs) could not be reliably captured due to variable prescription and over the counter use not well recorded in the dataset and therefore were not included as a study outcome. For children who did not complete 3 years of study, the reasons were noted and information up to the point of last study follow-up was included. The time to first sDMARD and first biologic among those who received these treatments was also determined from date of first rheumatology appointment to first drug start date within the treatment category.

To explore any changes to prescribing since the inception of CAPS, the cohort was divided into those recruited before and after January 1, 2006, the midpoint in the study recruitment. For all analyses, characteristics were compared across groups using non-parametric descriptive statistics, namely chi-squared and Kruskal–Wallis tests.

All analyses were performed using Stata, version 13 software (StataCorp., College Station, TX).

## Results

To January 1, 2011, 1137 children had been recruited to CAPS. We excluded 51 children with no ILAR assignment, nine with no recorded joint counts, 23 who withdrew consent and three with a diagnosis other than JIA, leaving 1051 children in this analysis (574 oJIA, 283 pJIA, 66 sJIA, 60 ERA and 68 PsA presentation) (Fig.).

Over the 3-year period, 137 had been lost to follow-up from the study and 169 had been discharged from paediatric rheumatology, for reasons including remission (40%), failing to attend appointments (26%) or transferring hospital (20%). The baseline characteristics across the groups are shown in Table 1.

### Treatment over the first 3 years following initial paediatric rheumatology appointment according to presenting disease pattern

#### Overall

Of 1051 patients included in this analysis, 86% received steroids in the first 3 years following presentation to rheumatology. Overall, 58% received sDMARD therapy and 20% received a biologic drug. The most commonly prescribed first-line biologic was etanercept (83%), then infliximab (9%) and adalimumab (5%). Three patients respectively were prescribed anakinra and tocilizumab first line with one prescribed canakinumab. There were 10% (*n* = 107) of patients for whom no use of steroids, sDMARDs or biologics in the first 3 years was recorded, including 55 who did not complete 3 years in the study. Of these 55, 27% were discharged well with 16% not attending clinic and 15% moving to other clinics. The median time from presentation to rheumatology to a patient starting sDMARD was 2 months [interquartile range (IQR): 0–8], with time to first biologic 14 months (IQR: 8–23).

#### Oligoarticular onset (oJIA)

A total of 574 patients presented with an oligoarticular pattern of disease. In all, 83% were treated with steroids (either intra-articular, oral or intravenous) over the first 3 years following presentation to paediatric rheumatology, and for 50% this was their only treatment (Table 2).

A total of 35% of oJIA patients (*n* = 199) received treatment with sDMARDs, most commonly methotrexate (92%). Median time from presenting to rheumatology to starting a first sDMARD was 9 months (IQR: 3–17). Overall, 55 oJIA patients (10%) received biologic therapy within 3 years, with a median time to first biologic of 23 months (IQR: 16–31). Etanercept was the most commonly prescribed first-line drug (67%), followed by infliximab (18%) and adalimumab (15%).

Patients with oJIA who received sDMARDs and biologics within 3 years had higher mean CHAQ, pain VAS and ESR scores at presentation to rheumatology than those treated with steroids alone (*p* < 0.05). Biologic treated patients were more likely to have a history of uveitis (biologic 30%, sDMARD 16% and steroid 5%). They were also more likely to be re-assigned to a more severe ILAR subtype within the first 3 years, most commonly extended oligioarthritis and polyarticular RF-negative JIA (Table 3). Oligoarticular patients with a history of uveitis that were treated with biologics were more commonly prescribed adalimumab and infliximab (*n* = 13) as opposed to etanercept (*n* = 4).

#### Polyarticular onset (pJIA)

There were 283 patients with polyarticular disease at first presentation (71% RF-negative pJIA, 12% RF-positive pJIA, 8% extended oligoarthritis and 9% undifferentiated JIA). In all, 94% of these patients also received treatment with sDMARDs, mainly methotrexate (98%). The median time from presentation to rheumatology to first sDMARD was less than 1 month (Table 2).

A total of 56% of pJIA patients received sDMARDs without receiving biologic therapy and 38% received biologics. The majority were treated with etanercept initially (92%), with a median time to first biologic of 13 months (IQR: 7–20). Six patients were treated with first-line infliximab, 2 tocilizumab and 1 starting adalimumab.

#### Systemic onset (sJIA)

Of the 66 patients presenting with systemic disease, 61 (93%) received treatment with sDMARDs, 59% of whom had also received steroids (Table 2). Over half of these patients had not received a biologic within 3 years. Median time to first sDMARD was 1 month (IQR: 0–3). Biologic therapy was prescribed in 30%, with a median time to first biologic of 10 months (IQR: 5–21). Etanercept was the most frequently prescribed first biologic (65%), three patients started anakinra, and one each started infliximab, adalimumab, tocilizumab and canakinumab.

#### Enthesitis-related arthritis onset (ERA)

A total of 60 patients presented with ERA and over half were treated with sDMARD therapy (Table 2). In all, 17% of patients were not recorded as receiving any steroids, sDMARDs or biologics over the first 3 years and 27% were treated with steroids only. There were 30% of patients that went on to receive biologic therapy; all but one started etanercept as their first treatment. The median time to starting a first biologic was 13 months (IQR: 10–19).

#### Psoriatic arthritis onset (PsA)

Of 68 patients presented with PsA of whom 74% were treated with sDMARD therapy, almost exclusively methotrexate (94%). Median time from presentation to rheumatology to first sDMARD was less than 1 month. Overall, 16% of patients (*n* = 11) received biologic therapy, with 9 (82%) starting etanercept as their first treatment. The median time to first biologic in those who received this therapy was 14 months.

### Treatment patterns by time

Patients presenting after January 1, 2006 were more often prescribed steroids (80% before versus 90% after 2006, *p* < 0.001) and sDMARDs (54% versus 61%, *p* = 0.02). Noted differences between disease groups included a higher use of sDMARDs in those presenting with ERA after January 1, 2006 (Table 4).

The median time to first sDMARD therapy was similar [overall before 2006: 3 months (IQR: 0–9); overall after 2006: 2 months (IQR: 0–8); *p* = 0.2]. There was a slight reduction in the time to first biologic, before 2006: 16 months (IQR: 9–28), after 2006: 14 months (IQR: 8–21), but did not reach statistical significance (*p* = 0.2). A higher proportion of patients were prescribed etanercept as a first-line biologic before 2006 (91% versus 78%), small but similar proportions presenting before/after January 1, 2006 received infliximab and anakinra, and the use of other biologics in those presenting after January 1, 2006 expanded in line with drug licensing.

## Discussion

This large, prospective study of non-trial based-clinical care describes the 3-year anti-rheumatic treatment patterns among children with JIA following presentation to paediatric rheumatology within one of five disease patterns, based on presenting active joint count and ILAR category. Among all children, 58% received a sDMARD and 20% received a biologic within this 3-year period, none of whom were prescribed abatacept over the period of observation. Rates of sDMARD use were highest in those presenting with polyarthritis and sJIA. However, 35% of those initially presenting with oligoarthritis later went on to receive sDMARDs and 10% received a biologic. These findings are not dissimilar from those reported recently from a Canadian JIA inception cohort, albeit over the first 5 years of disease in this latter study [10].

Patients presenting with an oligoarticular pattern of disease are typically treated initially with NSAIDs and intra-articular steroid injections rather than sDMARD and/or biologic therapy (3). The observation that many children with this presentation went on to receive sDMARDs and biologics may have been influenced by many factors. For some, an extension of their disease to extended oligoarthritis or polyarticular PsA or uJIA would have prompted a stepup in therapy. Our data also found that other factors related to the disease such as uveitis was also higher in those prescribed systemic therapy, although it was not known whether this was the main factor in the decision to treat. Adalimumab and infliximab were prescribed more frequently in oJIA patients with a history of uveitis, possibly influenced by reports of effectiveness of adalimumab and infliximab in treating uveitis, as well as less effective treatment and in some cases a worsening of uveitis in patients treated with etanercept [13]. There were, however, 60% sDMARD and 30% biologic treated oligoarthritis patients respectively who were still classified at the 3-year follow-up as having persistent oligoarthritis.

Despite a lack of clinical trial evidence for effectiveness in this category, persistent oligoarticular disease unresponsive to steroids may have prompted this decision to treat, particularly if persistent or damaging arthritis was present in certain critical joints (e.g., a dominant wrist and hip). Unfortunately, the granularity of data available in CAPS prevented a further investigation into this question.

Of patients with systemic arthritis, approximately one-third went on to receive a biologic therapy in the first 3 years. In the context of more severe disease, it might be expected that more children with sJIA would have been treated with biologics over this time period. However, this could be explained by a good level of disease control amongst this cohort with sDMARD alone. The majority of children with sJIA across the duration of our study were treated with TNFi as their first choice biologic rather than IL-1β and IL-6 inhibitors such as anakinra or tocilizumab [14]. However, for all but a minority of children, TNFi were the only available biologic choice over the first 3 years following presentation, with tocilizumab only licensed in 2011. A recent study from the British paediatric biologic registers have shown that since 2010, a majority of UK children with sJIA who are starting a biologic receive tocilizumab or anakinra as their first-line biologic therapy, although a majority do so after a trial of methotrexate [15].

The strengths of the study relate to the size of the cohort, the robust data collection methods employed and the prospective study design to minimise recall bias. However, this study is not without its limitations. It is an observational study capturing “real-world” data recorded during routine care. As such, missing data on both joint counts and ILAR categories were present, although a majority of children had an initial active joint count recorded.

We also noted that many children were discharged or lost-to-follow-up within the first 3 years following recruitment, which reflects the fact that many children will not remain under rheumatology care in the UK if they achieve drug-free remission. To reduce the potential of selection bias and maintain internal validity, the analysis included all patients recruited within the study period, including those who did not complete a full 3 years within the study. The majority of children who were discharged well had an oligoarticular presentation (77%). Therefore, excluding these patients may have overestimated the proportions of children requiring various therapies, particularly among the oligoarticular presentation group. It is accepted that we may also have underestimated the use of sDMARDs or biologics in those children who moved to a hospital, which was not participating in the CAPS study, as follow-up could not be completed. The analysis also consisted predominantly of patients diagnosed with JIA before the licensing of new biologics including tocilizumab. This may affect the generalisability of the data to a more modern JIA population, although this can be seen more in the choice of biologic rather than the decision to treat as all children presented within the biologic era. We did not see an increase in the use of biologics in the latter half of our study, only a wider range of biologics used.

To conclude, a high proportion of children presenting with JIA received sDMARDs and/or biologics during 3 years of follow-up, mainly in combination with or following initial steroid treatment. A high percentage of children initially presenting with oligoarthritis, many of whom were still classified as having persistent oligoarthritis at their 3-year follow-up, were also treated with biologic therapy. The use of biologics could in part be explained by a history of uveitis in some but not all of these patients. The effectiveness of biologic drugs in oligoarthritis is less well described. While there has been some research suggesting good methotrexate efficacy in persistent oligoarticular JIA [16], more research is needed to look at the effectiveness of both sDMARDs and biologics in the oligoarticular category to ensure the appropriate use of advanced therapies in this population. It would also be important to determine whether patient outcome is dependent on a treatment plan that is specific to JIA category or extra-articular manifestations versus level of active joints or disease pattern, for example, the prescription of IL-1β and IL-6 inhibitors in systemic JIA, which could serve to influence clinical decision making in the future.

## Figures and Tables

**Fig:**
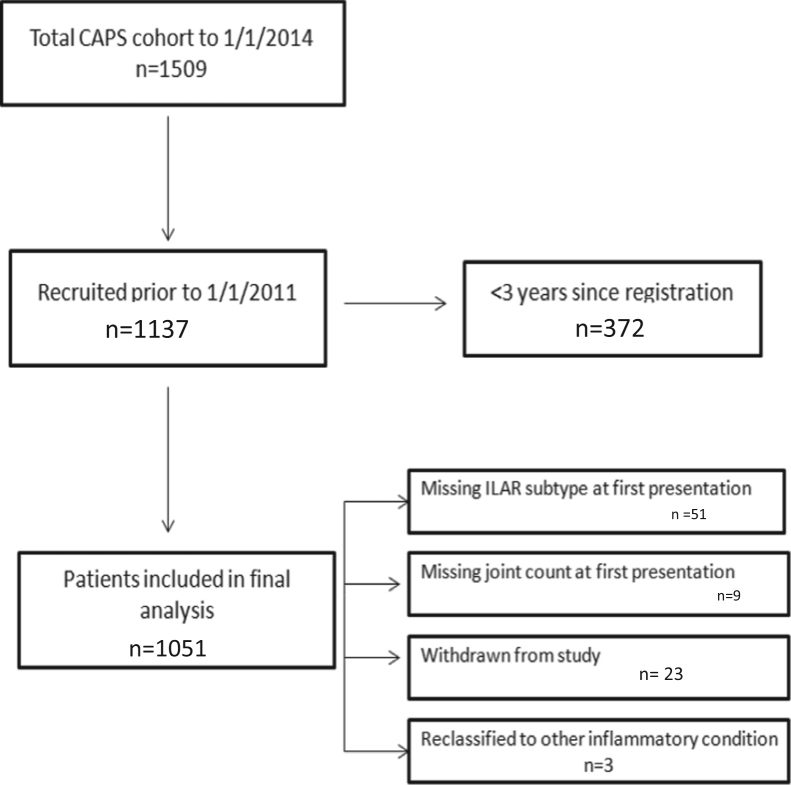
Study inclusion criteria.

**Table 1:** Patient Characteristics at first presentation to paediatric rheumatology

Characteristics	Overall cohort	oJIA	pJIA	sJIA	ERA	PsA	*p*
*N*	1051	574	283	66	60	68	
Age, median (IQR)	8 (3–12)	6 (3–10)	9 (4–12)	7 (4–11)	13 (11–14)	12 (7–13)	0.0001
Gender, % female	63	63	74	63	12	58	<0.0001
Disease duration (months), median (IQR)	5 (3–12)	5 (3–11)	6 (3–11)	2 (1–6)	8 (3–16)	8 (3–20)	0.0001
Active joint count, median (IQR)	2 (1–5)	1 (1–2)	8 (5–14)	3 (1–6)	2 (1–4)	3 (2–9)	0.0001
Limited joint count, median (IQR)	1 (1–3)	1 (1–2)	5 (2–10)	1 (0–5)	2 (1–4)	2 (1–5)	0.0001
ESR, median (IQR)	13 (6–32)	10 (5–25)	17 (8–39)	39 (10–84)	10 (5–25)	9 (5–25)	0.0001
CHAQ score, median (IQR)	0.5 (0–1.3)	0.3 (0–0.9)	0.8 (0.3–1.8)	0.6 (0.3–1.9)	0.1 (0–1.4)	0.8 (0.1–1.5)	0.0001
Pain VAS, median (IQR)	19 (2–49)	10 (1–39)	27 (5–56)	22 (9–64)	10 (0–33)	44 (7–64)	0.0001
Ever had uveitis, *n* (%)	82 (8)	55 (10)	19 (7)	0	5 (8)	3 (4)	0.071

Initial ILAR category, *n* (%)							
Systemic JIA	66 (6)	0	0	66 (100)	0	0	
Persistent oligoarthritis	531 (51)	531 (93)	0	0	0	0	
Extended oligoarthritis	22 (2)	0	22 (8)	0	0	0	
Polyarthritis RF −ve	201 (19)	0	201 (71)	0	0	0	
Polyarthritis RF +ve	34 (3)	0	34 (12)	0	0	0	
Enthesitis-related arthritis	60 (6)	0	0	0	60 (100)	0	
Psoriatic arthritis	68 (6)	0	0	0	0	68 (100)	
Undifferentiated arthritis	69 (7)	43 (7)	26 (9)	0	0	0	

**Table 2:** Anti-rheumatic treatment patterns overall and within disease subgroups

Drug pattern	Overall, *n* = 1051	Systemic, *n* = 66	Oligo, *n* = 574	Poly, *n* = 283	ERA, *n* = 60	PsA, *n* = 68
No steroid^a^, sDMARD or biologic treatment, *n* (%)	107 (10)	1 (2)	89 (15)	2 (1)	10 (17)	5 (7)
Steroid ever, *n* (%)	905 (86)	61 (92)	478 (83)	265 (94)	45 (70)	56 (82)
Steroid only, *n* (%)	332 (32)	3 (5)	285 (50)	15 (5)	16 (27)	13 (19)
sDMARD ever, *n* (%)	610 (58)	61 (93)	199 (35)	266 (94)	34 (56)	50 (74)
sDMARD only (i.e., no biologic), *n* (%)	398 (38)	41 (62)	144 (25)	158 (56)	16 (27)	39 (57)
sDMARD + steroid (no biologic treatment), *n* (%)	364 (35)	39 (59)	137 (24)	144 (51)	11 (18)	33 (49)
Median time from first presentation to rheumatology to first sDMARD [months (IQR)]	2 (0–8)	1 (0–3)	9 (3–17)	0.3 (0–3)	2 (0.2–4)	0.7 (0–6)
Biologic ever, *n* (%)	212 (20)	20 (30)	55 (10)	108 (38)	18 (30)	11 (16)
Median time from first presentation to rheumatology to first bio [months (IQR)]	14 (8–23)	10 (5–21)	23 (16–31)	13 (7–20)	13 (10–19)	14 (5–22)

a Steroids include intra-articular, oral, or intravenous treatment.

**Table 3:** Characteristics of oligoarthritis patients with differing treatment patterns

	Oligoarthritis patients treated with steroids^a^ only	Oligoarthritis patients treated with sDMARDs only (no biologics)	Oligoarthritis patients ever treated with biologics
*N*	285	144	55
Age, median (IQR)	7 (3–11)	5 (3–9)	6 (3–10)
Gender, % female	62	67	61
Disease duration (months), median (IQR)	5 (3–10)	5 (3–12)	4 (3–9)
Active joint count, median (IQR)	1 (1–2)	1 (1–2)	1 (1–2)
Limited joint count, median (IQR)	1 (1–2)	1 (1–2)	1 (1–2)
ESR, median (IQR)*	10 (4–20)	12 (6–34)	19 (6–40)
CHAQ score, median (IQR)*	0.1 (0–0.8)	0.5 (0–1.0)	0.8 (0–1.3)
Pain VAS, median (IQR)*	6 (0–29)	18 (2–42)	38 (7–62)
Median time from first presentation to rheumatology to first DMARD (months (IQR))		10 (3–24)	8 (3–15)
Uveitis recorded during follow-up, *n* (%)*	13 (5)	23 (16)	17 (30)

Last recorded ILAR subtype over 3 years of follow-up, *n* (%)*			
Systemic JIA	0	1 (1)	0
Persistent oligoarthritis	230 (81)	89 (62)	18 (33)
Extended oligoarthritis	4 (1)	25 (17)	20 (36)
Polyarthritis RF –ve	4 (1)	9 (6)	5 (9)
Polyarthritis RF +ve	1 (1)	1 (1)	2 (4)
Enthesitis-related arthritis	2 (1)	0	4 (7)
Psoriatic arthritis	4 (1)	4 (3)	1 (2)
Undifferentiated arthritis	9 (3)	1 (1)	1 (2)
Not recorded after first presentation	31 (11)	14 (9)	4 (7)

⁎ *p* < 0.05.

a Steroids include intra-articular, oral, or intravenous treatment.

**Table 4:** Anti-rheumatic treatment patterns overall and within disease subgroups among children recruited before and after January 1, 2006

		Overall	Systemic	Oligo	Poly	ERA	PsA
*N*	Before January 1, 2006	403	25	228	95	31	24
	After January 1, 2006	648	41	346	188	29	44

No steroid, sDMARD or biologic treatment, *n* (%)	Before	61 (15)	0	49 (21)	1 (1)	7 (23)	4 (17)
	After	46 (7)	1 (2)	40 (12)	1 (0.5)	3 (10)	1 (2)
	*p* Value	<0.001	0.4	0.001	0.6	0.2	0.03

Steroid ever, *n* (%)	Before	323 (80)	25 (100)	176 (77)	83 (87)	23 (74)	16 (67)
	After	582 (90)	36 (88)	302 (87)	182 (97)	22 (76)	40 (91)
	*p* Value	<0.001	0.07	0.002	0.02	0.9	0.01

sDMARD ever, *n* (%)	Before	216 (54)	24 (96)	78 (34)	86 (91)	12 (39)	16 (67)
	After	394 (61)	37 (90)	121 (35)	180 (96)	22 (76)	34 (77)
	*p* Value	0.02	0.4	0.9	0.08	0.004	0.3

sDMARD only (no biologic treatment), *n* (%)	Before	139 (34)	15 (60)	55 (24)	53 (56)	3 (10)	13 (54)
	After	259 (40)	26 (63)	89 (50)	105 (56)	13 (45)	26 (59)
	*p* Value	0.02	0.6	0.7	0.1	0.001	0.4

Median time from disease onset to first sDMARD, months (IQR)	Before	3 (0–9)	2 (0–3)	9 (4–18)	0.7 (0–5)	2 (0–11)	0.3 (0–1)
	After	2 (0–8)	0.5 (0–2)	9 (3–17)	0.2 (0–2)	2 (1–3)	1 (0–11)
	*p* Value	0.2	0.2	0.8	0.06	0.7	0.2

Biologic ever, *n* (%)	Before	77 (19)	9 (36)	23 (10)	33 (35)	9 (29)	3 (13)
	After	135 (21)	11 (27)	32 (9)	75 (40)	9 (31)	8 (18)
	*p* Value	0.4	0.6	0.8	0.4	0.9	0.5

Median time from disease onset to first biologic, months (IQR)	Before	16 (9–28)	8 (2–24)	27 (13–34)	15 (8–26)	16 (10–35)	18 (10–26)
	After	14 (8–22)	10 (7–16)	22 (18–27)	13 (7–20)	11 (10–19)	12 (4–18)
	*p* Value	0.2	0.9	0.5	0.3	0.6	0.5
